# B Cells Regulate Neutrophilia during *Mycobacterium tuberculosis* Infection and BCG Vaccination by Modulating the Interleukin-17 Response

**DOI:** 10.1371/journal.ppat.1003472

**Published:** 2013-07-11

**Authors:** Lee Kozakiewicz, Yong Chen, Jiayong Xu, Yanhua Wang, Kyri Dunussi-Joannopoulos, Qinglin Ou, Joanne L. Flynn, Steven A. Porcelli, William R. Jacobs, John Chan

**Affiliations:** 1 Department of Microbiology and Immunology, Albert Einstein College of Medicine, the Bronx, New York, New York, United States of America; 2 Department of Pathology, Albert Einstein College of Medicine, the Bronx, New York, New York, United States of America; 3 Immunology and Autoimmunity, Biotherapeutic Research, Pfizer, Cambridge, Massachusetts, United States of America; 4 Department of Immunology and Antibody Engineering, Genentech, San Francisco, California, United States of America; 5 Department of Microbiology and Molecular Genetics, University of Pittsburgh School of Medicine, Pittsburgh, Pennsylvania, United States of America; 6 Department of Medicine, Albert Einstein College of Medicine, Bronx, New York, United States of America; 7 Howard Hughes Medical Institute, Albert Einstein College of Medicine, the Bronx, New York, New York, United States of America; University of Massachusetts, United States of America

## Abstract

We have previously demonstrated that B cells can shape the immune response to *Mycobacterium tuberculosis*, including the level of neutrophil infiltration and granulomatous inflammation at the site of infection. The present study examined the mechanisms by which B cells regulate the host neutrophilic response upon exposure to mycobacteria and how neutrophilia may influence vaccine efficacy. To address these questions, a murine aerosol infection tuberculosis (TB) model and an intradermal (ID) ear BCG immunization mouse model, involving both the μMT strain and B cell-depleted C57BL/6 mice, were used. IL (interleukin)-17 neutralization and neutrophil depletion experiments using these systems provide evidence that B cells can regulate neutrophilia by modulating the IL-17 response during *M. tuberculosis* infection and BCG immunization. Exuberant neutrophilia at the site of immunization in B cell-deficient mice adversely affects dendritic cell (DC) migration to the draining lymph nodes and attenuates the development of the vaccine-induced Th1 response. The results suggest that B cells are required for the development of optimal protective anti-TB immunity upon BCG vaccination by regulating the IL-17/neutrophilic response. Administration of sera derived from *M. tuberculosis*-infected C57BL/6 wild-type mice reverses the lung neutrophilia phenotype in tuberculous μMT mice. Together, these observations provide insight into the mechanisms by which B cells and humoral immunity modulate vaccine-induced Th1 response and regulate neutrophila during *M. tuberculosis* infection and BCG immunization.

## Author Summary

*Mycobacterium tuberculosis* poses a serious threat to public health globally. It has been well established that T cells are critical in protection against *M. tuberculosis*. The role of B cells and humoral immunity in the process is less well understood. We previously showed that B cells and humoral immunity regulate the immune response against *M. tuberculosis*. The present study examined the mechanisms by which B cells regulate the host neutrophilic response upon exposure to mycobacteria and how neutrophilia may modulate the development of vaccine-induced protective immunity. The data reveal that B cells can regulate neutrophilia during *M. tuberculosis* infection and BCG vaccination by modulating the IL-17 response. Vaccination studies show that excess neutrophilia adversely affects the development of BCG-elicited Th1 response. These observations suggest that B cells can optimize the development of protective immunity upon BCG vaccination by regulating the IL-17/neutrophilic response. Understanding the mechanisms by which B cells and humoral immunity modulate the immune response during *M. tuberculosis* infection and BCG immunization, particularly those that regulate IL-17 levels and neutrophilia, may lead to the development of novel strategies for the control of the tubercle bacillus, including efficacious vaccines.

## Introduction

It has recently been demonstrated that B cells can shape the development of the immune response to *Mycobacterium tuberculosis* [1], [2]. The lungs of *M. tuberculosis*-infected B cell-deficient mice display exacerbated inflammation, with enhanced neutrophil recruitment [1]. Neutrophils are among the earliest cells to migrate to the site of *M. tuberculosis* infection and evidence exists that these phagocytes participate in the granulomatous reaction [3], [4]. Enhanced neutrophil infiltration has been associated with excessive lung pathology and with poor bacillary control in genetically susceptible mice [5], [6]. It has been proposed that neutrophilia is indicative of failed Th1 immunity in response to aerosol *M. tuberculosis* challenge [7]. There is also evidence suggesting that interaction of *M. tuberculosis* with neutrophils enhances DC migration to the draining lymph nodes thereby promoting the initiation of adaptive immune response in an aerogenic tuberculous infection [8]. Studies examining the significance of neutrophils in protection against *M. tuberculosis* have yielded conflicting results [3], [5], [9], [10], [11], [12], [13], [14], and the role of these professional phagocytes in TB remains to be clearly defined.

The cytokine IL-17 plays an important role in the recruitment of neutrophils to the site of inflammation [15], [16], [17], [18], including the airways, during infection [19], [20]. In autoimmune diseases and infection, IL-17 is produced by a variety of host cells, including myeloid cells [21], invariant natural killer (iNK) T cells [22], NK cells [23], [24], γδ T cells [25], [26], [27], and Th17 cells, a subset of helper CD4^+^ T lymphocytes [17], [28]. In a BCG immunization model, IL-17 produced by Th17 cells can downregulate IL-10 production and subsequently drives Th1 responses [29]. BCG vaccination induces Th17 cells that populate the lungs of immunized mice [30]. Upon challenge with *M. tuberculosis*, the Th17 cells recruit Th1 cells to the site of infection to restrict mycobacterial growth [30]. IL-17 can, however, promote tissue damage during *M. tuberculosis* infection [17], [31] and in the context of other infectious and autoimmune diseases [15], [16], [32], [33], [34]. It has been shown that repeated BCG vaccinations enhanced IL-17 production that is associated with increased neutrophil recruitment and exacerbated lung tissue pathology [35]. Therefore, a protective immune response against *M. tuberculosis* should promote Th17-mediated protection while mitigating the tissue damaging effects.

Ample evidence support the notion that B cells and the humoral immune response modulate T cell immunity [36], [37], including the development of memory T cell responses during infection [36], [37] and vaccine-induced protection against secondary challenge with intracellular pathogens such as Chlamydia [38] and Francisella [39]. Experimental evidence suggests that humoral immunity plays a role in regulating the Th1 response in TB [2]. Results derived from an X-linked immune-deficient (*xid*) B cell-deficient mouse model suggest that neutrophilia may adversely affect BCG vaccine efficacy [40]. Recent studies involving the B cell-depleting agent rituximab have revealed that B cells may enhance or diminish the IL-17/Th17 response [41], [42], [43], [44]. It has recently been reported that a subset of B cells in the blood of humans with tuberculous infection can suppress Th17 response [45].

These observations on B cells have prompted us to examine the role of B cells in regulation of IL-17 and neutrophilic responses to mycobacteria using an acute aerosol infection model as well as an ear ID BCG immunization model. The results provide evidence that B cells regulate neutrophilia during *M. tuberculosis* infection and BCG immunization by modulating the IL-17 response. The study also revealed that neutrophilia at the site of immunization adversely affects the development of BCG-induced Th1 response by diminishing DC migration to draining lymph nodes, thereby attenuating T cell immunity against *M. tuberculosis*. This latter observation suggests that B cells can optimize BCG-elicited Th1 immunity by regulating the IL-17/neutrophilic response. Finally, the lung neutrophilia and enhanced Th17 response seen in *M. tuberculosis*-infected B cell-deficient mice could be reversed by passive immune serum therapy, raising the possibility that immunoglobulins (Igs) may contribute to the regulation of these phenotypes.

## Results

### B cell-deficiency is associated with an augmented inflammation and neutrophilic infiltration in the lungs of *M. tuberculosis*-infected mice during the acute phase of infection

**Figure 1:**
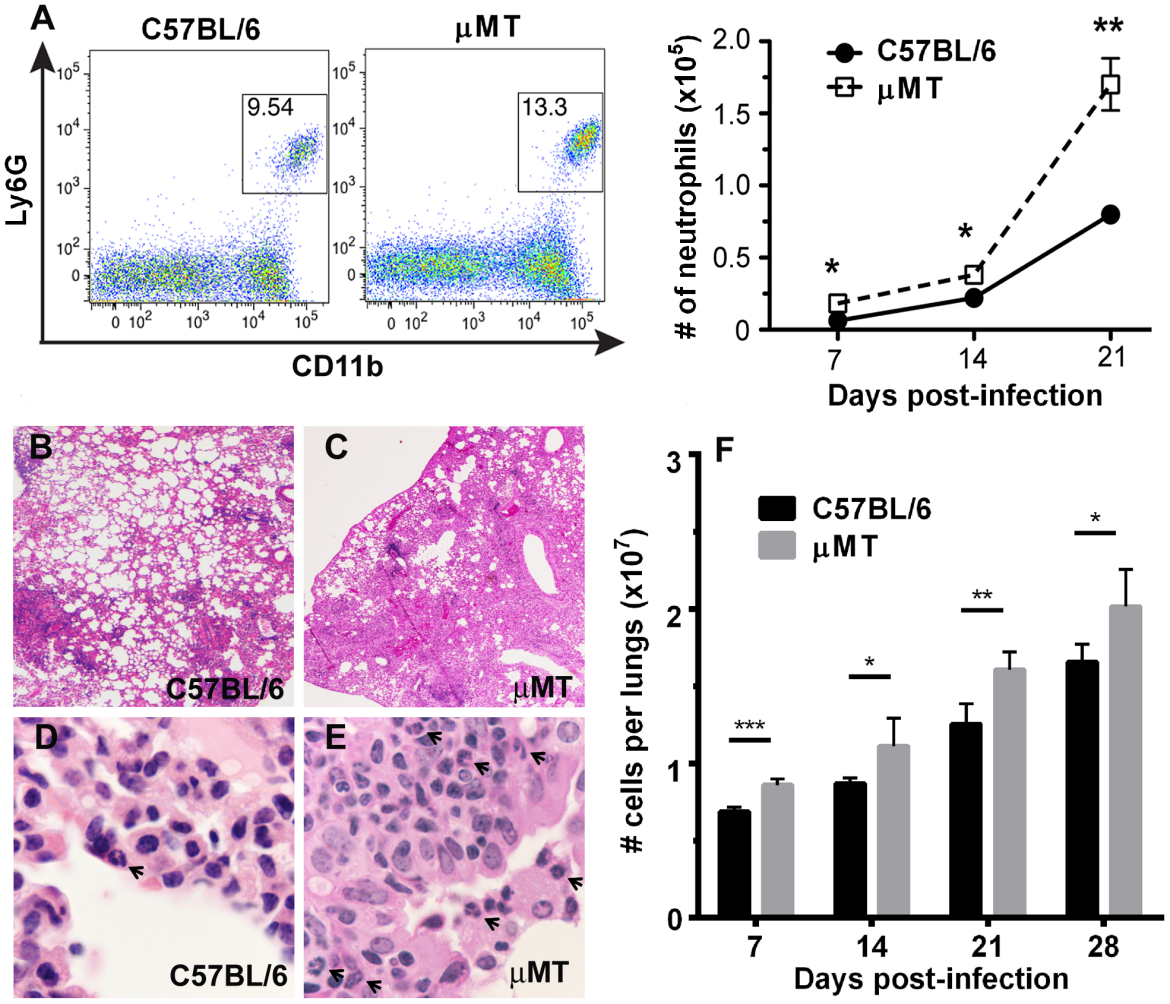
B cell-deficiency in μMT mice is associated with an augmented lung neutrophilic and inflammatory response in *M. tuberculosis*-infected mice during the acute phase of infection. Wild-type or B cell-deficient μMT mice were infected aerogenically with 200–300 CFU of *M. tuberculosis* Erdman. Lungs cells were procured for cytometric analysis, in conjunction with intracellular staining, at appropriate time intervals p.i.. ***A***, B cell-deficient μMT mice exhibited significant elevation, relative to wild-type animals, in the percentage and in the number of neutrophils (CD11b^+^Ly6G^+^) among lung cells during acute tuberculous infection. The FACS dot plot depicts the percentage of neutrophils at day 21 p.i.. (*p<0.05, **p<0.005). The number of neutrophils infiltrating the lungs was evaluated on day 7, 14, and 21 p.i.. The hematoxylin & eosin (H&E)-stained lung sections revealed increased level of total cellular (***B*** **&** ***C***; 2×) and neutrophilic (***D*** & ***E***; 100×) infiltration. During the first month p.i., total cells infiltrating the lungs increased gradually (**F**), at each time point studied, the number of cells in the lungs of μMT mice was significantly higher than that in WT's (**F**). The H&E sections represent lung tissues at 2 weeks p.i.. **Arrows:** neutrophils. ***p<.0001; **p<.001; *p<0.01. Data presented are representative of two to three experiments with three to five mice per group.

The aberrant immune response to *M. tuberculosis*, including excessive neutrophilia [1], has yet to be mechanistically explained. Emerging evidence suggest neutrophils play a significant role in modulating the immune response to *M. tuberculosis* [3], [4], [5], [6], [7], [8] We initiated studies to characterize the neutrophilic response in acute TB in the B cell-deficient μMT mouse [1]. Flow cytometric analysis of lung cells procured from *M. tuberculosis*-infected μMT mice revealed a neutrophilic response that was significantly higher than that observed in infected wild-type controls. At as early as 7 days post-infection (p.i.), the difference in lung neutrophilia between the two groups of mice was, albeit small, significant (p<0.05) (Figure 1A; Right Panel). By 21 days p.i., the absolute number of neutrophils in the lungs of μMT mice was over two fold that observed in wild-type animals (p<0.005). The proportion of neutrophils in the total lung cell population was also higher in tuberculous μMT mice relative to wild-type (Figure 1A; Left Panel & data not shown). This pulmonic neutrophilia of *M. tuberculosis*-infected μMT mice was also apparent histologically (Figure 1D&E), and was associated with an overall enhanced inflammatory response as assessed by histological examination of infected tissues (Figure 1B&C) and by quantifying the total number of cells infiltrating the lungs throughout the first month after infection (Figure 1F). Thus, the previously observed enhanced pulmonic neutrophil and exacerbated granulomatous inflammatory response of tuberculous μMT mice at 1 month p.i., when adaptive immunity sets in, is similarly observed in early acute TB, when the predominant anti-tuberculous response is mediated via innate immunity [1]. These data suggest that B cells can regulate the pulmonic inflammatory response, including that involving neutrophils, in the early acute phase of TB.

### Neutrophilia in the lungs of B cell-deficient mice with acute TB is associated with an enhanced IL-17/Th17 response

**Figure 2:**
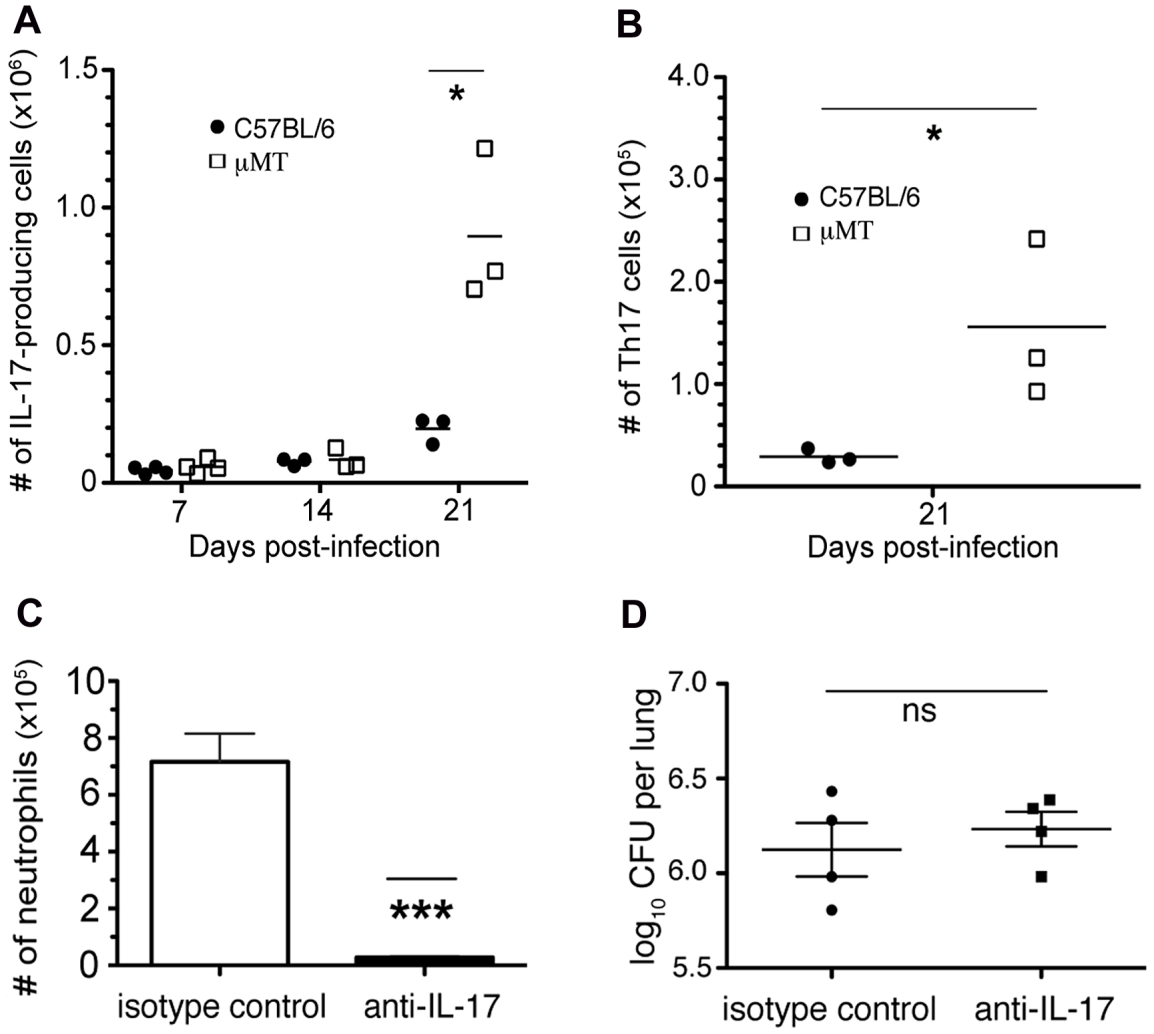
B cell-deficiency in μMT mice is associated with an augmented lung Th17 response in tuberculous mice during the acute phase of infection: reversibility of neutrophilia by IL-17 neutralization. Wild-type or B cell-deficient μMT mice were infected aerogenically with 200–300 CFU of *M. tuberculosis* Erdman. Lungs cells were procured for cytometric analysis, in conjunction with intracellular staining, at appropriate time intervals p.i.. ***A***, Compared to wild-type C57BL/6s, B cell-deficient μMT mice displayed increased in the number of IL-17A-producing lung cells and ***B***, total number of pulmonic Th17 cells (CD3^+^CD4^+^IL-17A^+^) upon *M. tuberculosis* infection (*p<0.05). ***C***, Quantification of neutrophil infiltration into the lungs of B cell-deficient μMT mice following IL-17 neutralization at day 14 p.i. revealed that increased neutrophilia was reversed via treatment with a mAb specific for this cytokine (***p<0.0005). ***D***, this reversibility was not due to differences in bacterial burden since the total numbers of CFU in the lungs of mAb-treated and -untreated mouse groups were comparable. Data presented are representative of two to three experiments with three to five mice per group.

The link between IL-17 and neutrophil recruitment [15], [16], as well as the observation suggesting a role for B cells and humoral immunity in modulating Th1 response in TB [2] and Th17 response in autoimmune diseases [41], [42], [43], [44], [45] prompted us to examine whether the neutrophilia observed in the lungs of μMT mice could be due to an aberrant Th17/IL-17 response as a result of B cell-deficiency. Flow cytometric analyses of lung cells, in conjunction with intracellular staining, revealed that on day 21 p.i., the numbers of IL-17-producing lung cells were elevated in tuberculous μMT mice compared to infected wild-type controls (Figure 2A), a subset of which is CD4^+^ Th17 cells (Figure 2B). The lung neutrophilia of tuberculous μMT mice could be significantly reversed by IL-17 neutralization (Figure 2C). Lung bacterial burden in μMT mice treated with IL-17 neutralizing antibodies (Abs) was comparable to those treated with isotype controls, excluding bacillary loads as a variable that may confound data interpretation (Figure 2D). These results suggest that IL-17, produced locally by lung cells including Th17 cells, plays a significant role in recruiting neutrophils to the lungs of B cell-deficient μMT mice in the early phase of acute *M. tuberculosis* infection and that B cells and humoral immunity play a role in regulating the IL-17/Th17 response in TB.

### B cell-depleted wild-type C57BL/6 mice also exhibit neutrophilia and augmented Th17 response in acute TB

**Figure 3:**
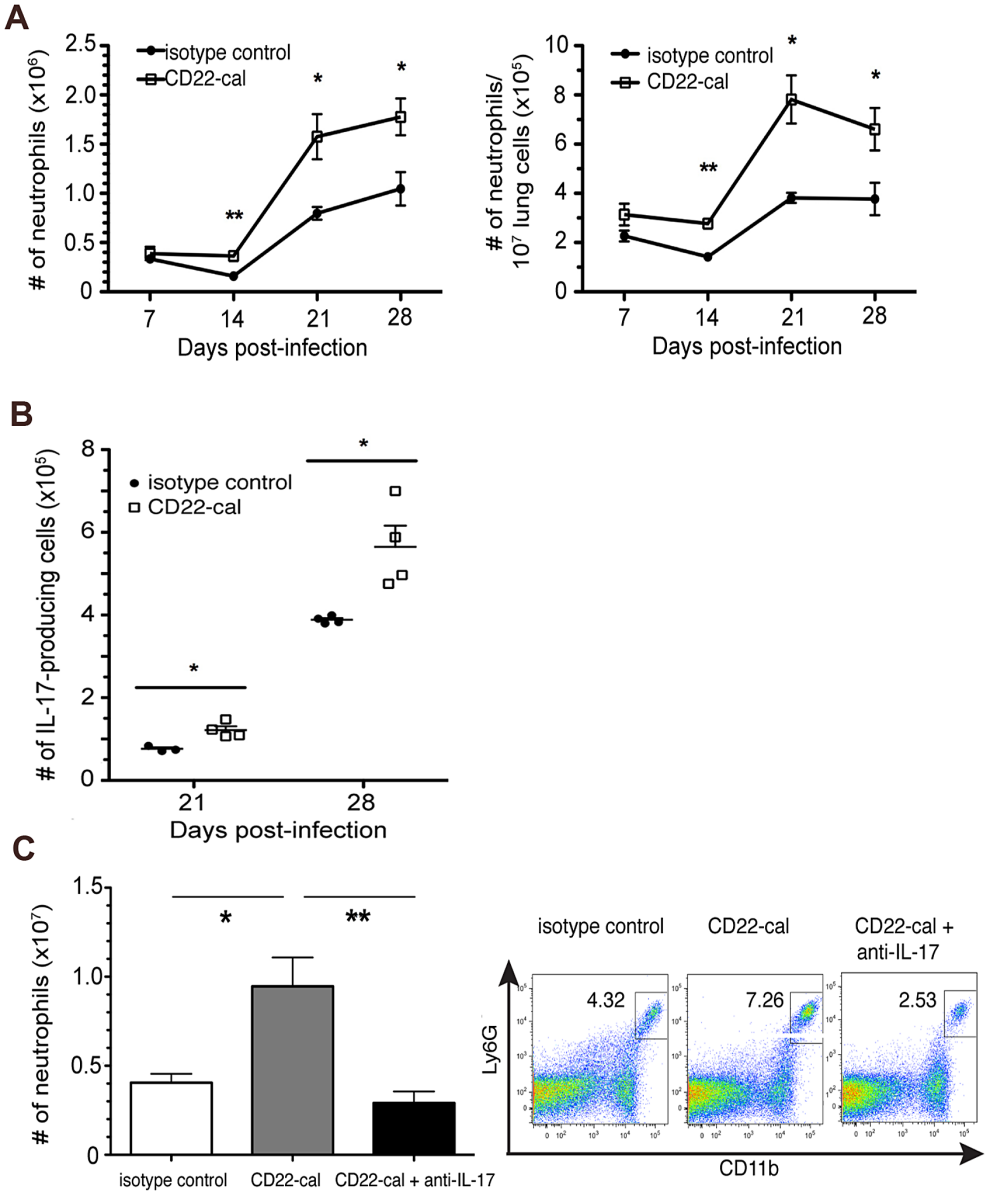
B cell depletion results in an increased neutrophilic and Th17 response in the lungs of C57BL/6 mice with acute TB: IL-17 neutralization reverses the neutophilia phenotype. Wild-type C57BL/6 animals or mice depleted of B cells using the CD22-cal conjugated Ab were infected aerogenically with 200–300 CFU of *M. tuberculosis* Erdman; lung cells were prepared at appropriate time intervals after infection and subjected to intracellular staining and/or flow cytometric analysis. Administration of CD22-cal resulted in ∼95% depletion of CD19+ B cells in the lungs of *M. tuberculosis*-infected mice at day 21 p.i. (see Figure S1A). ***A***, The proportion and absolute number of neutrophils (CD11b^+^Ly6G^+^) among total lung cells in mice rendered B cell-deficient by CD22-cal treatment are elevated relative to those detected in non-B cell-depleted animals (*p<0.05, **p<0.005). ***B***, CD22-cal-treated B cell-depleted mice, relative to untreated wild-type animals, exhibited a significant increase in total IL-17-producing lung cells during the acute phase of tuberculous infection (*p<0.05). ***C***, Neutralization of IL-17 in CD22-cal-treated mice reverses lung neutrophilia phenotype associated with B cell-depletion at day 14 p.i. (*p<0.05, **p<0.005). Data depicted are representative of two to three experiments with three to five mice per group.

The previously reported B cell deficiency-associated phenotypes, which include lung neutrophilia at 1 month after aerogenic *M. tuberculosis* challenge [1], were observed in the μMT strain rendered B cell-deficient by targeted disruption of the membrane exon of the μ chain gene [46]. That these observations are B cell-specific is strongly supported by reversal of the B cell deficiency phenotypes by adoptive B cell transfer [1]. Nevertheless, to rigorously test the B cell-specificity of the Th17/IL-17/neutrophilia phenotype observed in the μMT strain in acute TB (Figures 1&2), we conducted experiments involving B cell depletion via administration of CD22-cal, an effective B cell-depleting agent [47], [48] (Figure S1A), in wild-type C57BL/6 mice. In agreement with the results of the μMT studies, neutrophilic infiltration in the lungs of tuberculous B cell-depleted C57BL/6 mice was higher compared to that observed in undepleted controls (Figure 3A). Neutrophilia in B cell-depleted mice was apparent as early as 7 days after aerogenic challenge, and increased steadily over the next 2 weeks, peaking at day 21 to day 28 post-inoculation (Figure 3A). This neutrophilia was associated with an increase in IL-17-producing lung cells (Figure 3B). As in the μMT study, IL-17 neutralization reversed the neutrophilia phenotype in *M. tuberculosis*-infected, B cell-depleted mice (Figure 3C). The congruency of the B cell-depletion and μMT studies strongly support that the IL-17/Th17/neutrophila phenotype is B cell-specific, supporting a role for B cells in regulating neutrophilia in acute TB through modulation of the IL-17 response.

### B cell deficiency results in a diminished BCG vaccine-induced Th1 response in mice

**Figure 4:**
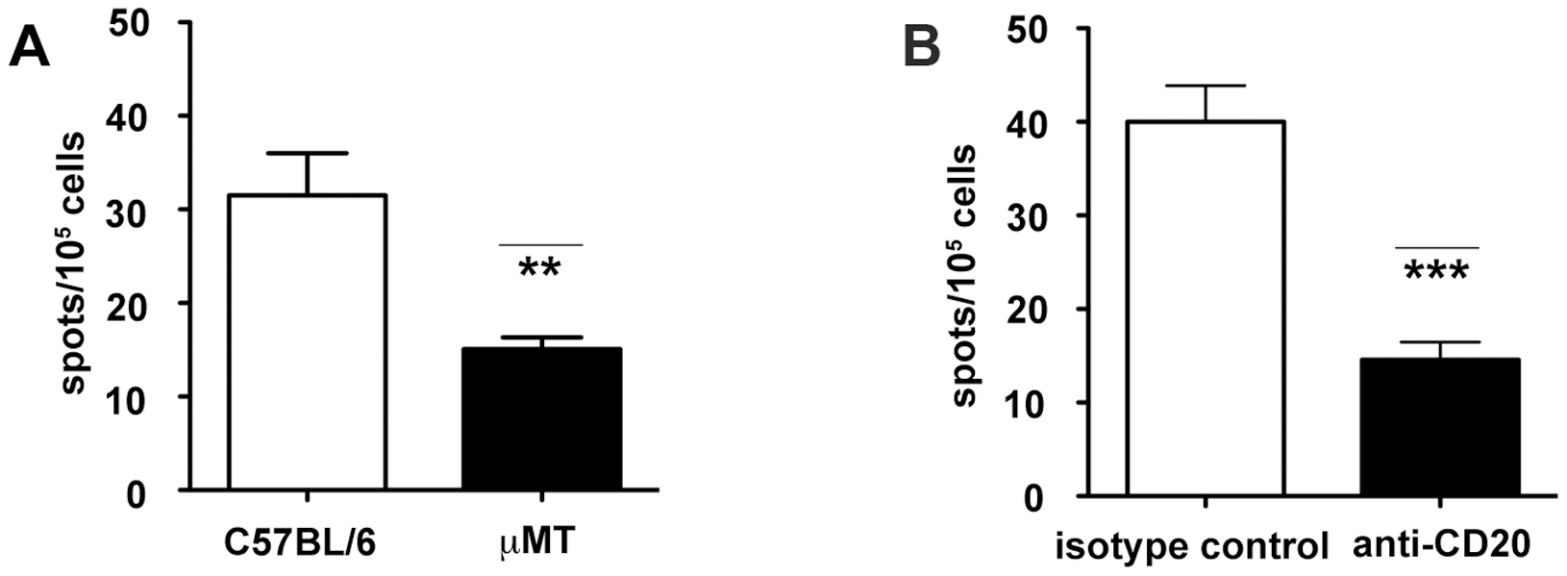
B cell deficiency results in a diminished Th1 response in BCG-immunized mice. Wild-type or B cell-deficient mice were vaccinated with BCG and at one month post-immunization, splenic T cells were assessed for the development of Th1 response by quantification of IFN-γ-producing CD4^+^ T cells. ***A***, ELISPOT analysis of the number of splenic P25-Ag85B-specific IFN-γ-producing CD4^+^ T cells in wild-type and μMT mice 1 month after vaccination revealed a diminished Th1 response in the B cell deficient group (** p<0.005). ***B***, A similarly decrease in BCG-induced Th1 response was also observed in mice rendered B cell-deficient via treatment with the anti-CD20 5D2 mAb (***p<0.0005). Data shown are representative of three independent experiments with four mice per group.

It has been suggested that neutrophilia in the B cell-deficient *xid* mouse may diminish anti-TB vaccine efficacy, although the B cell-specificity of the phenotypes and the mechanisms underlying the enhanced neutrophilic response was not examined [40]. This observation, together with the Th17/neutrophilia phenotype observed in *M. tuberculosis*-infected B cell-deficient mice (Figures 1–3), led us to hypothesize that B cells and humoral immunity, by virtue of their ability to modulate the IL-17 response and neutrophilia (Figures 1–3) upon exposure to mycobacteria, may affect the efficacy of anti-TB vaccine. To begin testing this hypothesis, we assessed the effects of B cell deficiency on the BCG-induced Th1 response in mice. The results showed that upon subcutaneous (SC) BCG immunization, B cell-deficient mice, compared to wild-type mice, developed a significantly attenuated Th1 response (Figure 4A). This attenuated Th1 response was also apparent in C57BL/6 mice treated with 5D2 (Figure 4B), a highly effective B cell-depleting anti-CD20 Ab (Figure S1B). Together, the μMT and the 5D2 vaccination studies have provided compelling evidence that B cells are required for optimal development of BCG-induced Th1 response.

### Characterization of the effect of B cell-deficiency on the BCG-induced Th1 response: The ID ear vaccination mouse model

**Figure 5:**
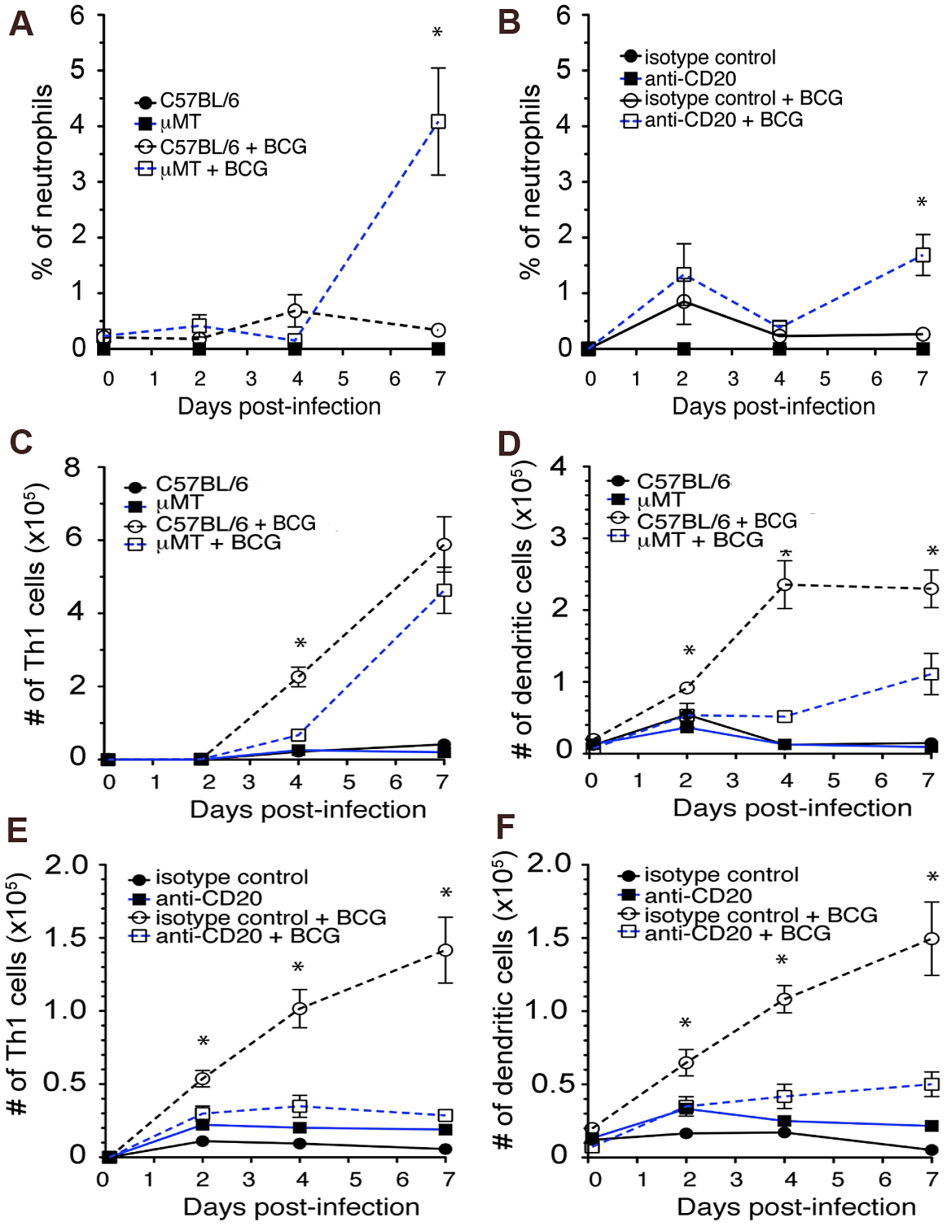
B cell-deficient mice, upon ID ear immunization with BCG, display an enhanced neutrophilic infiltration in the pinna and an attenuated Th1 response in the draining superficial cervical lymph nodes that are associated with diminished DC migration. Ears of wild-type or B cell-deficient mice were vaccinated intradermally with BCG and assessed at the indicated time intervals post-immunization. Cells from the ear and the draining superficial cervical lymph nodes were procured as described in Materials and Methods and subjected to flow cytometric analysis. μMT mice (***A***) and C57BL/6s depleted of B cells using the 5D2 anti-CD20 mAb (***B***) exhibited elevated numbers of CD11b^+^Ly6G^+^ neutrophils compared to wild-type animals (*p<0.05). Results of flow cytometric analyses showed a diminished number of IFN-γ-producing CD4^+^ Th1 cells (***C***) and CD11c^+^ DC (***D***) in draining superficial cervical lymph nodes of B cell-deficient μMT mice (relative to wild-type C57BL/6's) that were immunized intradermally in the ear with BCG (1×10^6^ CFU) (*p<0.05). This Th1 (***E***) and DC migration (***F***) phenotypes were similarly observed in mice depleted B cells via treatment of the 5D2 anti-CD20 mAb (*p<0.05). Data shown are representative of two to three independent experiments with four mice per group.

**Figure 6:**
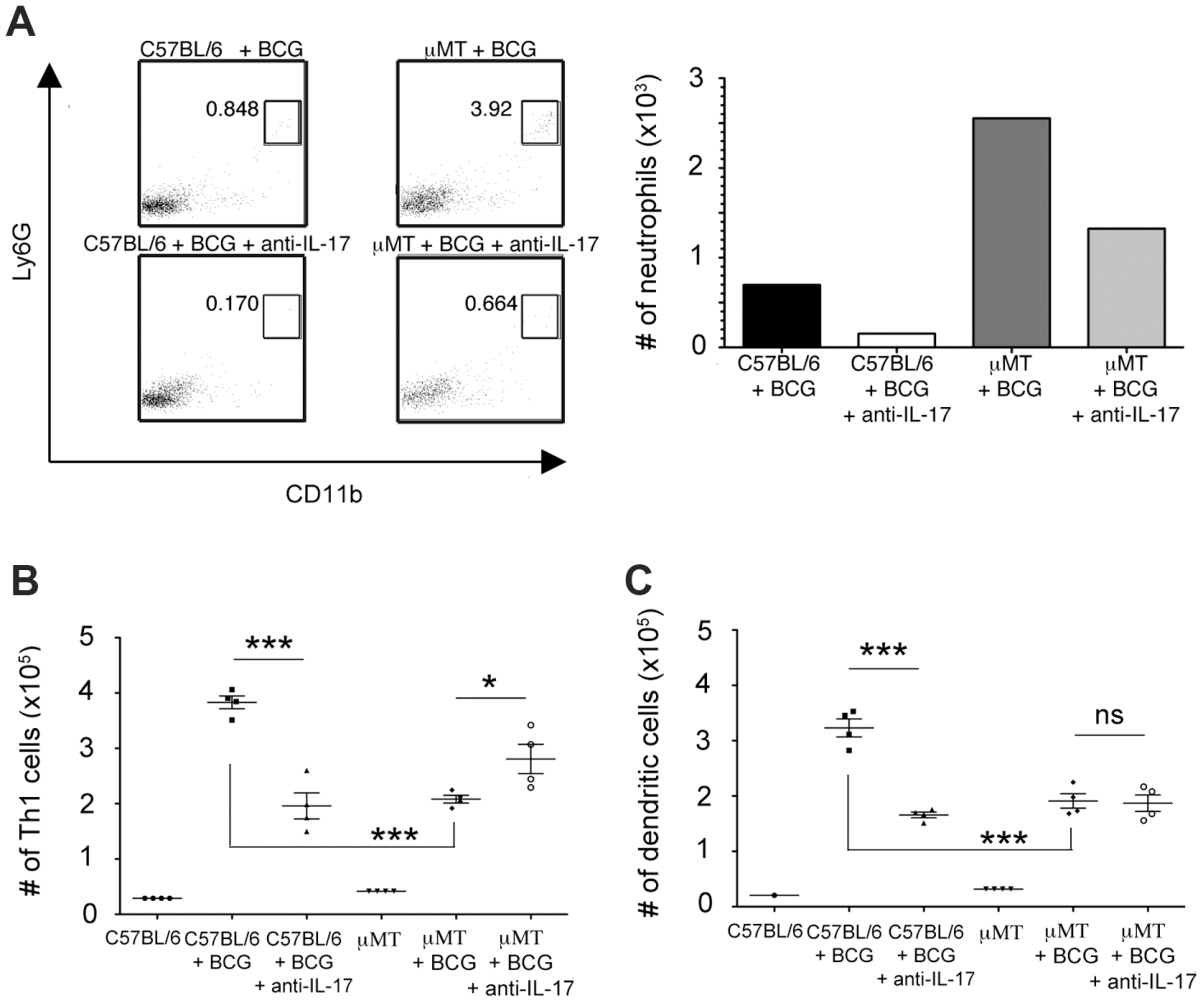
Effect of IL-17 neutralization on the level of neutrophilic infiltration in the ear and on the DC migration to and the Th1 response in the draining cervical lymph nodes of mice following ID BCG ear immunization. ***A***, The frequency (Left Panel) and absolute number (Right Panel) of neutrophils (CD11b^+^Ly6G^+^) in the ear dermis of B cell-deficient μMT mice on day 7 following BCG vaccination was increased relative to that observed in wild-type C57BL/6 animals. In these studies, cells obtained from the vaccinated ears were pooled for analysis. The enhanced pinna neutrophilic response was attenuated upon IL-17 neutralization. Upon ID ear vaccination, the B cell-deficient μMT mice exhibited decreased number of IFN-γ-producing CD4^+^ Th1 cells (***B***) and CD11c^+^ DC cells (***C***) in the draining superficial cervical lymph nodes of μMT mice relative to those detected in wild-type C57BL/6's. While IL-17 neutralization increased the number of Th1 cells (***B***) in the draining cervical lymph nodes of μMT mice intradermally immunized in the ear, it had no apparent effects on the number of DC (***C***). By contrast, IL-17 neutralization attenuated DC migration (***C***) to and the Th1 response (***B***) in the draining cervical nodes of wild-type C57BL/6 mice (*p<0.05, ***p<0.0005). The data are representative of two independent studies with four mice per group.

A limitation of the SC BCG immunization mouse model, which involves administration of the vaccine at the scruff of the neck of the animal, is the variability in the anatomic location of the lymph nodes to which immune cells (e.g., DCs) migrate from this site of vaccination. In addition, it is difficult to locate the precise area of vaccination to procure immune cells for analysis. These problematic features of the SC immunization model complicate the analysis of local immunological events that ensue upon immunization. Consequently, we adopted an ear ID vaccination model to circumvent this hindrance, since the anatomical location of the draining lymph nodes of the ear (the superficial cervical lymph nodes) is well defined, and the site of immunization in the pinna can be readily localized [49]. In this model, the level of neutrophil infiltration was higher in the ears of vaccinated μMT mice relative to that detected in wild-type animals as early as 7 days post-vaccination (Figure 5A). The number of neutrophils at the site of vaccination was also higher in immunized μMT mice relative to wild-type mice (Figure 6A, Right Panel). Neutrophilia was similarly observed in BCG vaccinated sites in the ear of mice rendered B cell-deficient via 5D2 treatment (Figure 5B). These data revealed that in B cell-deficient mice, pulmonic neutrophilia observed during the acute phase of *M. tuberculosis* infection occurs also at the site of ID BCG vaccination in the ear.

Since B cell deficiency is associated with sub-optimal development of BCG-elicited Th1 response (Figure 4), and since DC priming of CD4^+^ T cells is critical to the development of vaccine-induced protection, we initiated studies to characterize the kinetics of DC migration to the draining lymph nodes following BCG vaccination. The results revealed that μMT or B cell-depleted C57BL/6 mice exhibited diminished numbers of CD11c^+^ DCs in the superficial cervical lymph nodes compared to wild-type controls starting at as early as day 2 and continuing through day 7 post-vaccination (Figure 5D&F). The BCG-induced Th1 response of the two B cell-deficient mouse groups, as assessed by the number of interferon (IFN)-γ-producing CD4^+^ T cells in the draining superficial cervical lymph nodes, was diminished compared to that detected in vaccinated wild-type animals (Figure 5C&E). These data suggest that B cell deficiency results in impaired T cell priming following BCG immunization. The decreased number of BCG-induced DC migrated to the draining lymph nodes in the B cell-deficient animals could be a factor that contributes to the impairment of CD4^+^ T cell priming observed in the μMT and B cell-depleted mice.

### The effects of IL-17 neutralization on the development of BCG-induced Th1 response in B cell-deficient mice

The enhanced pinna neutrophilia and the impaired Th1 response observed in B cell-deficient mice upon BCG immunization (Figure 5) prompted us to investigate the relationship between these two observations. Based on the finding that lung neutrophilia in *M. tuberculosis*-infected mice is Th17/IL-17 driven (Figures 2 & 3), we first examined the effect of IL-17 neutralization on BCG-induced Th1 response in the ear immunization model. In agreement with results obtained from the lung infection model (Figures 2 & 3), IL-17 neutralization significantly attenuated the BCG-induced neutrophilic response in the ear inoculation model, reducing the number of neutrophils in the BCG-vaccination site of C57BL/6 and B cell-deficient animals by 78% and 49%, respectively (Figure 6A). The results indicate that IL-17 is involved, at least in part, in neutrophil recruitment to the site of BCG immunization in both B cell-deficient and B cell-sufficient states.

IL-17 neutralization modulates BCG-elicited Th1 response in wild-type and B cell-deficient μMT mice, albeit in opposing fashion (Figure 6B). Concomitant with the decrease in neutrophilia at the site of immunization upon IL-17 neutralization, there was an increased BCG-elicited Th1 response in the B cell-deficient mice (Figure 6B). By contrast, IL-17 neutralization in immunized wild-type C57BL/6 mice resulted in a diminished Th1 response (Figure 6B). The incongruent effects of IL-17 neutralization on the BCG-induced Th1 response among wild-type and μMT mice were also apparent on DC migration to the draining cervical lymph nodes following vaccination (Figure 6C). IL-17 neutralization during BCG immunization of μMT mice did not alter the number of DCs in the draining cervical lymph nodes 7 days post-immunization, but decreased that in wild-type mice (Figure 6C). These data suggest that i) IL-17 modulates the BCG-elicited Th1 response; and ii) the enhanced IL-17/Th17 response in B cell-deficient μMT mice upon BCG immunization contributes to the diminished vaccine-elicited Th1 immunity. This effect could be mediated by IL-17/Th17 per se and/or through the level of neutrophilic infiltration at the site of vaccination. The discrepant effects of IL-17 neutralization on vaccination-induced Th1 response in wild-type C57BL/6 and μMT mice warrant further investigation.

### Neutrophil depletion improves DC migration to and priming of Th1 cells in draining lymph nodes

**Figure 7:**
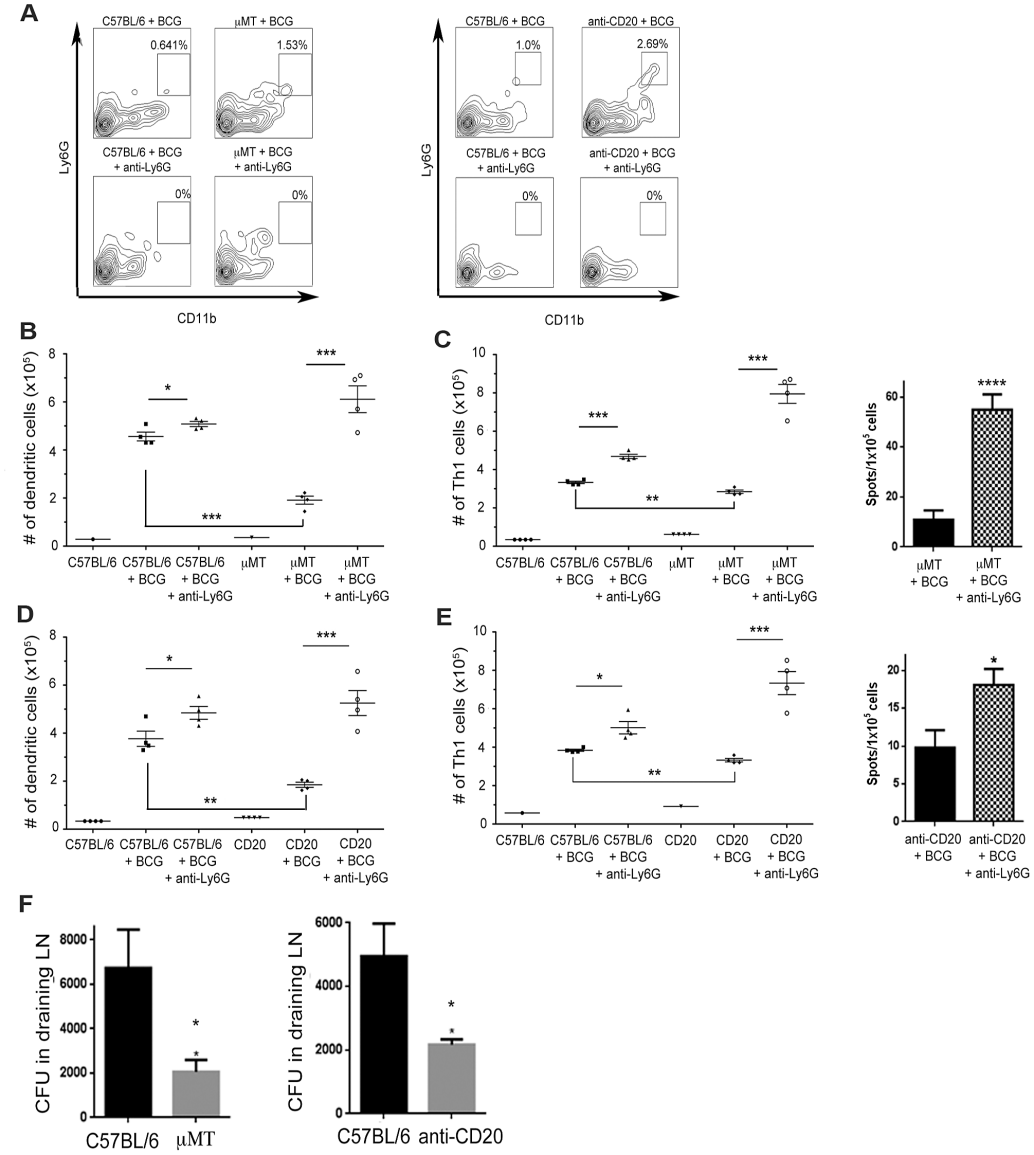
Neutrophil depletion in wild-type and B cell-deficient mice results in enhanced DC migration to and an increased Th1 response in the draining superficial cervical lymph nodes upon ID ear BCG immunization. C57BL/6 wild-type and μMT B cell-deficient mice, as well as animals depleted of B cells by treatment with the 5D2 anti-CD20 mAb were vaccinated with 10^6^ CFU of BCG at the ear intradermally. Lymph nodes cells were procured at day 7 post-immunization and subjected to flow cytometric and IFN-γ ELISPOT assay analyses (using the Ag85B P25 peptide as antigens). ***A***, μMT mice (Left Panel) and C57BL/6s depleted of B cells using the 5D2 anti-CD20 mAb (Right Panel) exhibited elevated numbers of CD11b^+^Ly6G^+^ neutrophils at the site of ID ear BCG vaccination compared to wild-type animals. The neutrophilia in both groups of mice was abrogated upon treatment with the Ly6G-specific mAb 1A8. 1A8 depletion of neutrophils resulted in increase in the numbers of CD11c^+^ DC (***B***) and IFN-γ-producing CD4^+^ T cells (***C***, Left Panel: intracellular staining; Right Panel: Ag85B P25 peptide-specific ELISPOT assay) in the draining cervical lymph nodes of both the ear BCG-vaccinated μMT and wild-type C57BL/6 mice (*p<0.05, **p<0.005, ***p<0.0005). Neutrophil depletion with 1A8 led to similar enhancement of DC migration (***D***) to and Th1 response (***E***: Left Panel: intracellular staining; Right Panel: Ag85B P25 peptide-specific ELISPOT assay) in the cervical draining lymph nodes post ID BCG vaccination in the ear of both wild-type and 5D2-treated B cell-deficient mice (*p<0.05, **p<0.005, ***p<0.0005). ***F***, CFU enumeration in the draining superficial cervical lymph nodes at 7 days post-BCG immunization revealed decreased bacterial load in the μMT mice and animals depleted of B cells using the 5D2 anti-CD20 mAb (*p<0.05, ****p<0.00005). The results shown are representative of two independent experiments. Four mice per group were used in each experiment.

IL-17 neutralization resulted in only partial attenuation of the neutrophilic response in the ear vaccination model (Figure 6A). Due to this partial effect and the link between IL-17 and neutrophilic recruitment, the IL-17 depletion approach does not allow clear determination of the relative contribution of this cytokine and neutrophils to the observed B cell-deficiency-associated Th1 and DC phenotypes. To begin to address this issue, we conducted neutrophil depletion experiments using the Ly6G-specific Ab, clone 1A8 [14]. 1A8 completely depleted neutrophils at the site of BCG immunization of both wild-type C57BL/6 and μMT mice (Figure 7A). Neutrophil depletion reverses the DC and Th1 phenotypes in BCG-vaccinated μMT mice (Figure 7B&C). Treatment with 1A8 increased the number of IFN-γ-producing CD4^+^ T cells in the draining lymph nodes of μMT mice on day 7 post-immunization by over two fold (Figure 7C). Neutrophil depletion increased the number of DCs in the draining lymph nodes of the μMT mice by three fold (Figure 7B). Similar enhancing effects of neutrophil depletion on the number of Th1 cells and DCs were observed in the draining lymph nodes of BCG-vaccinated C57BL/6 mice (Figure 7B&C), albeit to a lesser extent than μMTs. To stringently test the B cell-specificity of these observations, we examined the effects of neutrophil depletion on C57BL/6 mice rendered B cell deficient using the anti-CD20 Ab 5D2. Results derived from the 5D2-based model are similar to those obtained using the μMT mice (Figure 7D&E).

These results strongly suggest that in the ear vaccination model, B cells contribute significantly to the development of vaccine-induced Th1 immunity by modulating the IL-17 driven neutrophilic response. In the absence of B cells, neutrophils play a dominant role in down-regulating the development of vaccine-induced Th1 response. The mechanisms by which neutrophils adversely affect the development of BCG-induced Th1 response remained to be defined. One possibility is the ability of neutrophils to internalize BCG, thereby competing with DCs (the primary antigen presenting cell (APC) responsible for the initiation of the anti-TB immune response) for antigens, which could result in a sub-optimal antigen dose required for T cell priming in lymph nodes. In support of this notion, bacterial load in the draining cervical lymph nodes of B cell-deficient μMT and 5D2-treated mice is lower than that of wild-type C57BL/6 mice (Figure 7F).

### Immune serum therapy in μMT mice reverses the neutrophil and Th17 phenotypes

**Figure 8:**
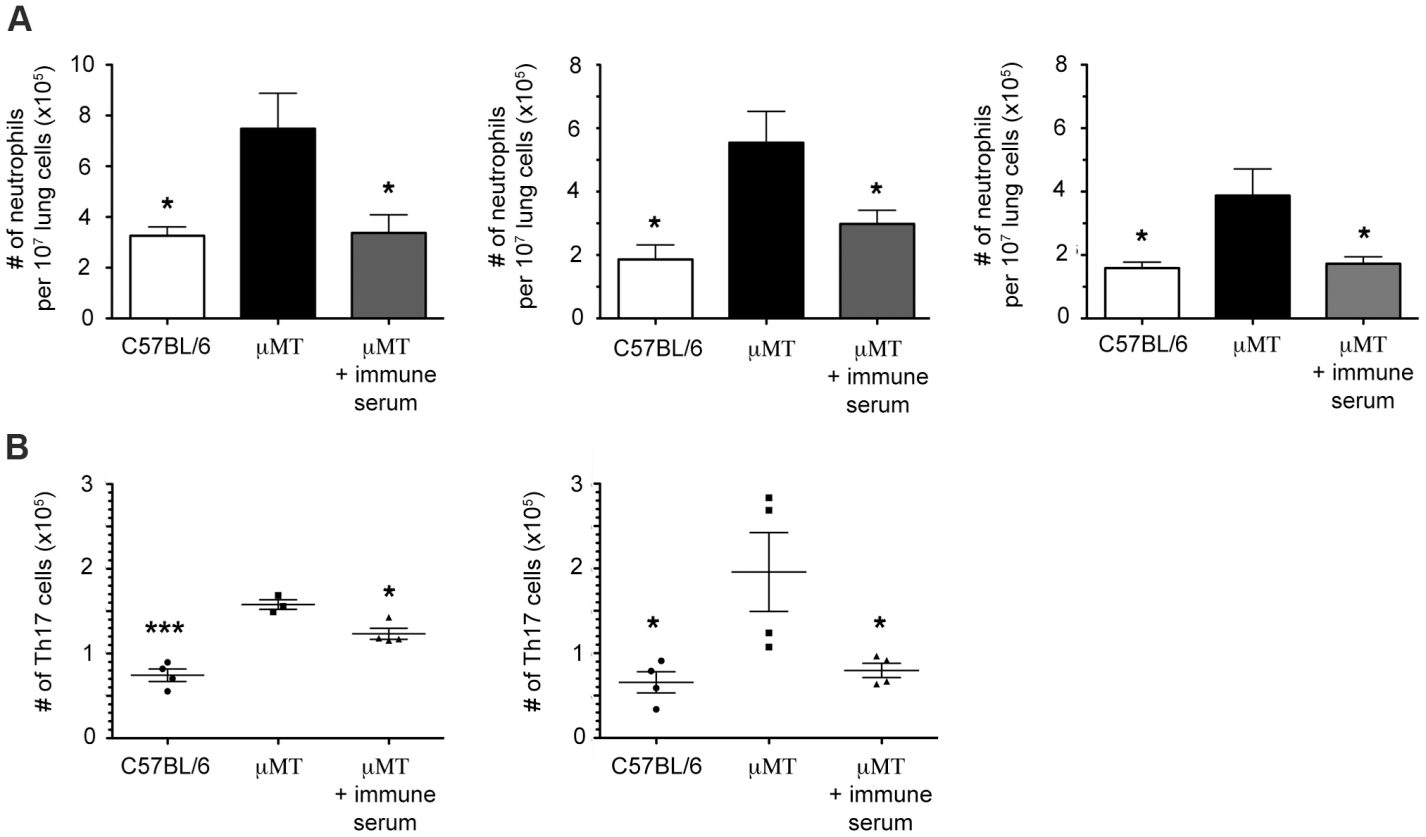
Passive transfer of immune serum into B cell-deficient mice reverses the neutrophil and Th17 phenotypes. B cell deficient μMT mice were aerogenically infected with 100–300 CFU of *M. tuberculosis* Erdman and lung neutrophilia assessed at 1 month p.i.. Mice were treated with immune sera produced from tuberculous wild-type C57BL6 mice at one month post-aerosol infection, Passive transfer of immune serum from infected wild-type C57BL/6 mice into tuberculous μMT mice resulted in diminished lung neutrophil infiltration (***A***, * p<0.05) and Th17 response (***B***, *p<0.05, **p<0.005, ***p<0.0005) at 1 month p.i.. The results of three neutrophil and two Th17 experiments are shown. Four mice per group were used in all experiments.

Previously we have shown that adoptively-transferred B cells (derived from *M. tuberculosis*-infected wild-type C57BL/6 mice) into μMT mice with TB effectively reverses the lung phenotypes associated with B cell-deficiency (including neutrophilia) during acute TB [1]. That the transferred B cells are able to reverse pulmonic phenotypes of infected μMT mice without homing to the lungs suggests regulation via a soluble factor(s). In that same study, the transfer of B cells resulted in the appearance of Igs in the recipient μMT mice [1]. These results suggest the possibility that Igs may be one factor that can reverse the B cell-deficiency phenotypes, including neutrophilia. In support of this possibility, results generated from an *M. tuberculosis* infection model involving FcγR knockout mouse strains strongly suggest that humoral immunity can regulate host responses (including tissue neutrophilia) to the tubercle bacillus [2]. To begin testing the Ig hypothesis, we conducted serum therapy study, which revealed that treatment of tuberculous B cell-deficient μMT mice with immune sera derived from *M. tuberculosis*-infected wild-type animals reversed the lung neutrophilia phenotype (Figure 8A). This serum therapy also resulted in a decrease in the number of Th17 cells in the lungs of treated tuberculous μMT mice (Figure 8B). The data suggest that components of the immune serum, possibly Igs, may play a role in regulating the neutrophil and Th17 response during acute *M. tuberculosis* infection.

## Discussion

Cell-mediated immunity is well established as critical in defense against *M. tuberculosis* [50], [51], [52]. By contrast, the significance of B cells and humoral immunity in shaping the immune response to the tubercle bacillus is less clear [37], [53], [54]. Studies using B cell-deficient μMT mice, in conjunction with B cell transfer, have provided evidence that B cells and the humoral immune response can modulate anti-tuberculous immunity, including the level of granulomatous inflammation and neutrophilic infiltration in lungs [1]. FcγR knockout mouse studies suggest that humoral immunity can influence development of Th1 responses during *M. tuberculosis* infection [2]. The data of the present study add the IL-17/Th17/neutrophils axis to the list of immunological factors regulated by B cells and humoral immunity in TB.

The results generated in two different models (lung *M. tuberculosis* infection and the ear ID BCG vaccination) involving μMT and C57BL/6 mice rendered B cell-deficient via treatment with two independent B cell-depleting agents, revealed B cell-dependent tissue neutrophilia at the site of interaction between mycobacteria and the host. These observations, together with data yielded by the IL-17 neutralization and neutrophil depletion experiments, provided strong evidence that i) B cells regulate neutrophilia during *M. tuberculosis* infection and BCG immunization; ii) exuberant early neutrophilia can adversely affect vaccine-induced Th1 response by impairment of DC migration to draining lymph nodes, thereby compromising CD4^+^ T cell priming; and as a result, iii) B cells can optimize BCG-elicited Th1 immunity by regulating the IL-17/neutrophil response. Neutrophilia in the lungs of tuberculous μMT mice is apparent as early as 7 days p.i., which is prior to an increase in the number of total lung IL-17-producing cells (observed on day 21 post-aerosolization), suggesting the possibility that IL-17-independent mechanisms are involved in the regulation of B cell dependent lung neutrophilia [55]. The decrease in the number of DCs in the draining lymph nodes of vaccinated B cell-deficient mice suggests that excess neutrophilia at the site of vaccination can result in competition between neutrophils and DCs for BCG, thereby affecting vaccine dosage and likely DC maturation and hence migration to the lymph nodes. This notion is supported by the decrease in bacterial burden in the draining lymph nodes of immunized B cell-deficient mice. It is, however, possible that the antigen uptake and migration capacity of the DCs at the site of immunization of a B cell-deficient host, is adversely affected, directly or indirectly, by the excessive neutrophilia (independent of the BCG competition theory) that occurs in the dermis of the vaccinated ear. The results that IL-17 neutralization with concomitant decrease in neutrophilia improves Th1 response in BCG-vaccinated μMT mice without affecting the number of lymph node DCs suggest the possibility that the immunization site in the ear of the μMT mice provides a local environment, of which excessive neutrophilia is a feature, can qualitatively compromise the capacity of DCs to effectively prime CD4^+^ T cells.

The opposing effects of IL-17 neutralization on BCG-elicited Th1 response in vaccinated wild-type and μMT mice are intriguing. The results generated by the IL-17 neutralization experiments involving wild-type mice are, in fact, in agreement with a previous study reporting that IL-17 can drive BCG-elicited Th1 response [29]. The discrepant effects of IL-17 neutralization observed among wild-type and μMT mice in the BCG vaccination model could be secondary to differences in the level of the IL-17 response and to the dissimilarities in the quantity and quality of neutrophils at the immunization site among the two mouse groups, with the level of neutrophilia in the ear dermis of B cell-deficient μMT strain being significantly greater than that detected in the C57BL/6 wild-type mice. These same variables could result in the differential effect of IL-17 neutralization on the number of DCs in the draining lymph nodes of the two mouse groups. It will be of interest to determine the functional properties of the neutrophils infiltrating the immunization sites of the μMT and wild-type mice. Collectively, the IL-17 neutralization studies have provided evidence that B cells can optimize BCG-induced Th1 immunity by modulating the IL-17/neutrophil response.

Results of the neutrophil depletion experiment support a role for these cells in regulating development of Th1 responses during vaccination. It has been reported recently that neutrophils enhance CD4^+^ T cell priming in the early phase of an aerogenic *M. tuberculosis* lung infection by promoting DC migration to draining mediastinal lymph nodes [8]. In the ear ID BCG immunization model employed here, neutrophil depletion enhances the vaccine-induced Th1 response. The discrepant data regarding the role of neutrophils in modulating CD4^+^ T cell priming could be due to the obvious multiple variables in the two models employed, including but not limited to the differences in the strains of mycobacteria studied (BCG versus *M. tuberculosis*), in the quality of the DCs (lungs versus skin), and in the conditions in which the mycobacteria interact with the host (lungs parenchyma versus dermis). In a *Leishmania major* model involving ID ear infection of metacyclic promastigotes, neutrophil depletion also results in enhanced priming of pathogen-specific CD4^+^ T cells [56].

By contrast and worthy of note, the observation that IL-17-neutralized C57BL/6 mice displayed a decrease in BCG-induced Th1 response and in the number of DCs in draining lymph nodes lends indirect support to the recently reported role for neutrophils in promoting T cell priming in acute TB [8], since IL-17 neutralization decreases the number of neutrophils at the site of immunization in the ear vaccination model. These contrasting observations of the IL-17 neutralization and the neutrophil depletion studies could be due to differences in the immunological environment resulting from IL-17 neutralization versus neutrophil depletion. For example, IL-17 neutralization results in partial decrease in the degree of neutrophilia at the site of immunization and in the level of IL-17, while 1A8 treatment results in virtually complete depletion of neutrophils without apparent interference with the amounts of IL-17. Together, the results also suggest that the complex roles of neutrophils in development of immune response to *M. tuberculosis* could depend on the characteristics of the site of immunological reaction, the level of neutrophilia as well as the interaction with other immune molecules. Understanding the mechanisms underlying the differential effects of neutrophil depletion on the development of the Th1 response in the lung infection and ear immunization model may shed light on the requisites for the development of optimally efficacious anti-TB vaccines.

The precise mechanisms by which B cells regulate the IL-17 response remain to be determined. The effects of IVIG (intravenous immunoglobulins) treatment on *M. tuberculosis*-infected mice [57] and results obtained from the FcγRIIB- and Fcγ chain-deficient mouse TB models strongly support a role for Igs in regulating anti-TB immunity, including the Th1 response [2]. The notion is further supported by the ability of adoptively transferred B cells from infected C57BL/6 mice to reverse neutrophilia in the lungs of infected μMT mice without having to home to the lungs [1]. In the current study, we demonstrate that immune sera from infected mice can reverse the lung neutrophilia and Th17 phenotypes in B cell-deficient mice with TB. It is thus tempting to speculate that Igs, known effective modulators of inflammation [53], [58], are a factor in the immune sera with the capacity to ameliorate the B cell-deficiency associated neutrophilia and Th17 phenotypes. We are cognizant of the complex nature of sera and as such, much work needs to be done in order to establish or refute a role for Igs in regulating the neutrophilic response in a tuberculous host. Experiments designed to address this issue are currently underway.

In summary, results of the present study provide strong evidence that B cells can regulate tissue neutrophilia during *M. tuberculosis* infection and BCG vaccination by modulating the IL-17 response. In addition, the study has provided evidence that the compromised BCG-induced Th1 response associated with B cell-deficiency is secondary to neutrophilia. These observations suggest that B cells can optimize BCG-elicited Th1 immunity by regulating the IL-17/neutrophilic response. It appears that optimally effective immunization protocols should achieve a balanced level of IL-17/neutrophilic response, which can only be achieved through a comprehensive understanding of the biology of these two immunological elements. Elucidation of the mechanisms by which B cells regulate the IL-17/neutrophilia response in *M. tuberculosis*-host interaction may help the design of novel effective TB vaccines and of protocols for the treatment of inflammatory disorders. Last but not least, the overall congruent results of the μMT and B cell depletion experiments suggest the validity of the use of the μMT mouse to investigate the role of B cells and humoral immunity in the development of anti-mycobacterial immune responses [1].

## Materials and Methods

### Ethics statement

Animal studies were conducted in accordance to the National Institutes of Health guidelines in compliance with assurance of the well being of laboratory animals. All protocols used in the study have been approved by the Institutional Animal Care and Use Committee of Albert Einstein College of Medicine (protocol # 20110602).

### Animals

Female C57BL/6 mice (Charles River Laboratories) and B cell-deficient mice (μMT; Jackson Laboratories) [46], 8–10 weeks old, were used in all experiments. Infected mice were housed in our biosafety level-3 laboratory and kept pathogen-free by routine serological and histopathological examinations. Animal protocols employed in this study were approved by the Institutional Animal Care and Use Committee of Albert Einstein College of Medicine.

### Mycobacteria, mouse infection, and determination of tissue bacterial burden

Bacterial stocks of *M. tuberculosis* strain Erdman (Dr. Frank Collins, Trudeau Institute, Saranac Lake, NY) were generated by passaging through mice to maintain virulence as previously described [1]. Bacteria were stored at −80°C in aliquots until use. Mice were infected with 100–300 colony forming units (CFU) of *M. tuberculosis* via aerosol inhalation using a Glas-Col inhalation chamber to deliver the desired inoculum [59]. For each experiment, the accuracy of inoculum dose was confirmed by assessing the number of bacterial colonies upon plating serial dilutions of whole lung homogenates on 7H10 agar plates (Difco) as previously described [1]. Tissue bacterial burden was quantified as previously described by plating serial dilutions of lung, liver, and spleen homogenates onto 7H10 agar plates at appropriate intervals after infection [1]. Tissue bacterial load was determined by counting the number of colonies on 7H10 agar plates after incubation at 37°C for 4 to 6 weeks.

### Preparation of single-cell suspension from tissues

Single cell suspensions were prepared as previously described [1]. Briefly, aseptically procured lungs, spleen, or lymph nodes were dissected at appropriate times after exposure to mycobacteria, then minced using sterile razor blades (Fisher Scientific) in cold RPMI 1640 with L-glutamine supplemented with 25 mM HEPES, 10% fetal bovine serum (FBS; GIBCO), and 55 µM 2-mercaptoethanol (complete RPMI). Minced lung tissues were then incubated in 1 mg/mL collagenase and 30 µg/mL DNase (Sigma Aldrich) at 37°C for 30 min. Digested tissues were passed through a 70-µm pore nylon cell strainer (Falcon; BD Biosciences) using the flat end of a sterile syringe plunger. The resultant cell suspensions were then treated with ACK lysis buffer (Invitrogen) to lyse red blood cells. Cells were then washed twice with complete RPMI. The method of trypan blue exclusion was used to enumerate live cells. For the ID model of immunization, ear dermal sheets were split along the cartilage and placed skin-side up in RPMI complete supplemented with 10% FCS and collagenase (1 mg/mL), followed by an one-hour incubation at 37°C and 5% CO_2_ to prepare suspensions of cells. Single cell suspensions were obtained by filtering through a 70-µm cell strainer (BD Falcon).

### Flow cytometry and intracellular staining

Single cell suspensions prepared from various tissues as described above, were washed in Flow Cytometry Staining Buffer (eBioscience). Flow cytometric analysis and intracellular staining was conducted as previously described [1], [2]. For each sample, approximately 10^6^ cells were suspended in Flow Cytometry Staining Buffer. Cellular Fcγ receptors were blocked by incubating cells in Fc block (BD Biosciences) on ice for 10 min. Cells were then immunostained for flow cytometric analysis with the following fluorochrome-conjugated Abs: anti-CD4-PE-Cy7 (clone RM4-5), anti-CD11c-FITC (clone HL3), anti-CD11b-PE-Cy7 (clone M1/70), anti-Ly6G-FITC (clone 1A8), B220-PerCP (clone RA3-6B2), CD22-PE (clone Cy34.1), CD19-FITC (clone 1D3), anti-IFN-γ-PE (clone XMG1.2), and anti-IL-17A-PE (clone TC11-18H10), all from BD Pharmingen; and anti-CD3-APC (clone 145-2C11; eBioscience). Dead cells were excluded from analysis using the LIVE/DEAD Fixable Violet Dead Cell Stain Kit (Invitrogen) according to manufacturer's protocol. Samples were collected on an LSRII (BD Biosciences, San Jose, CA), and data analysis was performed using FlowJo software (TreeStar, Ashland, OR). To stain for intracellular cytokines, single lung cell suspensions prepared as described above were stimulated in wells of 96-well plates in RPMI-1640 with 10% (vol/vol) FBS for 2 hours at 37°C and 5% CO_2_ in the presence of PMA (50 ng/mL) and ionomycin (500 ng/mL). Brefeldin A (1 µg/mL; eBioscience) was then added to the wells, and incubation was continued for another 2 hours at 37°C and 5% CO_2_. Following stimulation, the FoxP3 staining buffer kit (eBioscience) was used according to the manufacturer's protocol for intracellular cytokine staining.

### B cell depletion

For B cell depletion studies, two different reagents were used to deplete this lymphocytes population from wild-type C57BL/6 mice. The first reagent, CD22-cal (Pfizer), is an immunoconjugate consisting of a monoclonal antibody (mAb) that reacts specifically with mouse CD22 (a B cell-specific surface molecule), conjugated to N-acetyl-calicheamicin dimethyl acid, an enediyne anti-tumor agent [47], [48] that mediates dose-dependent cytotoxicity upon internalization [60], [61]. CD22-cal was administered at a dose of 160 µg/kg intraperitoneally (i.p.) at 12 and 7 days before *M. tuberculosis* infection, and then at 9 and 14 days post-inoculation. This regimen has been shown in various mouse models to effectively deplete B lymphocytes without affecting other immune cells [47]. This CD22-cal protocol routinely attains ∼95% of splenic B cell-depletion in treated mice at the time of *M. tuberculosis* infection and subsequent time intervals of analysis (Figure S1A and data not shown). 5D2, an anti-CD20 mAb (Genentech), was another B cell-depleting agent used. Mice received a dose of 10 mg/kg of Ab intravenously two days prior to ID ear or SC BCG immunization. Two weeks after the initial dose, anti-CD20 5D2 was then given i.p. at a dose of 5 mg/kg every other week for maintenance. This 5D2 protocol was shown in pilot experiments to be highly effective in B cell-depletion in mycobacteria-infected mice (Figure S1B).

### The BCG immunization models

BCG immunization was administered either subcutaneously or intradermally. For the conventional SC vaccination model, 10^6^ BCG Pasteur were inoculated subcutaneously in the scruff of the back of the neck in 100 µl of PBS containing 0.05% Tween-80 (Sigma Aldrich). For the ID vaccination model, an inoculum of 10^6^ BCG (determined in standardization experiments to be the optimal dose), in 10 µl of PBS containing 0.05% Tween-80 (Sigma Aldrich), was injected intradermally into the ear pinna of anesthetized mice using a Hamilton Microlitre syringe. Single cell suspensions of splenocytes and of the pinna and draining lymph nodes were obtained as described above and subjected to the IFN-γ ELISPOT assay (see below) and flow cytometric analysis.

### IFN-γ ELISPOT assays

The IFN-γ ELISPOT assay was used to assess the CD4^+^ T cell response upon exposure to mycobacteria as previously described [62]. T cells were purified from appropriate tissues at the desired time intervals using the Mouse Pan T Cell Isolation Kit II (Miltenyi Biotech). Detection of IFN-γ-producing T cells was conducted using the Mouse IFN-γ ELISPOT Ready-Set-Go Kit (eBioscience) according to manufacturer's instructions. Briefly, T cells (1×10^5^ and 3×10^5^) were seeded in wells of 96-well ELISPOT plates (Millipore) that had been coated overnight with IFN-γ capture Ab. Peptide 25 (aa 240–254) of *M. tuberculosis* antigen 85B (P25-Ag85B) was used to stimulate CD4^+^ T cells [62]. Splenocytes from naïve uninfected mice were used asAPCs. The APCs were pulsed with P25-Ag85B (10 µg/mL) for 1 hour at 37°C and 5% CO_2_, washed twice, and added to T cell-seeded wells (2×10^5^ cells per well). T cells co-cultured with unpulsed APCs served as controls. After a thirty-six-hour incubation at 37°C and 5% CO_2_, cells were removed and the captured cytokine was detected using a biotinylated anti-mouse IFN-γ Ab (clone R4-6A2; eBiosciences). Avidin-horseradish peroxidase (eBioscience) was added to the wells for 45 minutes at room temperature and colorization was achieved using AEC substrate solution (eBioscience). The substrate reaction was stopped by washing the plate with distilled water. Spots were enumerated using an automated ELISPOT reader.

### In vivo IL-17 neutralization

Two anti-mouse IL-17 mAb's were used. One was obtained from R & D systems (50104; IgG_2_) and another from Genetech (IgG_1_; Gift of Dr. Wenjun Ouyang, Genentech). The mAb's were administered was administered i.p. at a dose of 100 µg per mouse (in 200 µl of sterile PBS) every 3–4 days starting one day before *M. tuberculosis* infection or BCG immunization for the duration of the study. This regimen has been shown to effectively neutralize IL-17 in various experimental models [63]. Control groups received matched IgG isotypes.

### In vivo neutrophil neutralization

For neutrophil depletion, the anti-mouse Ly6G Ab (BioXcell; clone 1A8) or Rat IgG_2_ a isotype control (BioXcell; clone 2A3), 200 µg per mouse in 100 µl of sterile PBS, was administered i.p. every 3–4 days as previously described [64] starting one day before BCG immunization for the duration of the study.

### Passive serum transfer experiments

Blood was collected retro-orbitally from C57BL/6 mice infected with 100 CFU *M. tuberculosis* Erdman at 1 month p.i.. Sera were collected after clarification by centrifugation of clotted blood and stored at −80°C until use. Sera thus collected are referred to as immune sera. One hundred µl of immune sera were administered i.p. every 3–4 days into μMT mice, starting 1 day before infection with 100–300 CFU of *M. tuberculosis* Erdman, and continued for 1 month thereafter. At 1 month p.i., lung cells were procured from mice and subjected to intracellular staining and flow cytometric analysis.

### Statistical analysis

Where appropriate, the statistical significance of data points was determined using the unpaired Student *t* test or ANOVA analysis, assessed using GraphPad Prism 5 software. A *p* value of <0.05 was considered significant.

## Supporting Information

Figure S1 **B cell depletion in C57BL/6 mice using CD22-cal and the 5D2 anti-CD20 Abs.** ***A***, Administration of CD22-cal according to the protocol described in Materials and Methods resulted in ∼95% depletion of CD19^+^ B cells in the lungs of *M. tuberculosis*-infected mice at day 21 p.i.. ***B***, Administration of the 5D2 anti-CD20 mAb according to the protocol described in Materials and Methods led to effective B cell depletion (∼97% depletion) at the start of *M. tuberculosis* aerogenic infection of C57BL/6 mice that was maintained for the duration of the experiment (4 weeks p.i.). Results shown are representative of 2 independent experiments with 3 mice in each experimental group. WT: wild-type C57BL/6 mice treated with isotype control Abs.
(TIF)
