# Supplementary figures and images for: B Cells Regulate Neutrophilia during Mycobacterium tuberculosis Infection and BCG Vaccination by Modulating the Interleukin-17 Response
Source: PLoS Pathog. 2013 Jul 11;9(7):e1003472. doi: 10.1371/journal.ppat.1003472 (PMC3708864; doi:10.1371/journal.ppat.1003472)

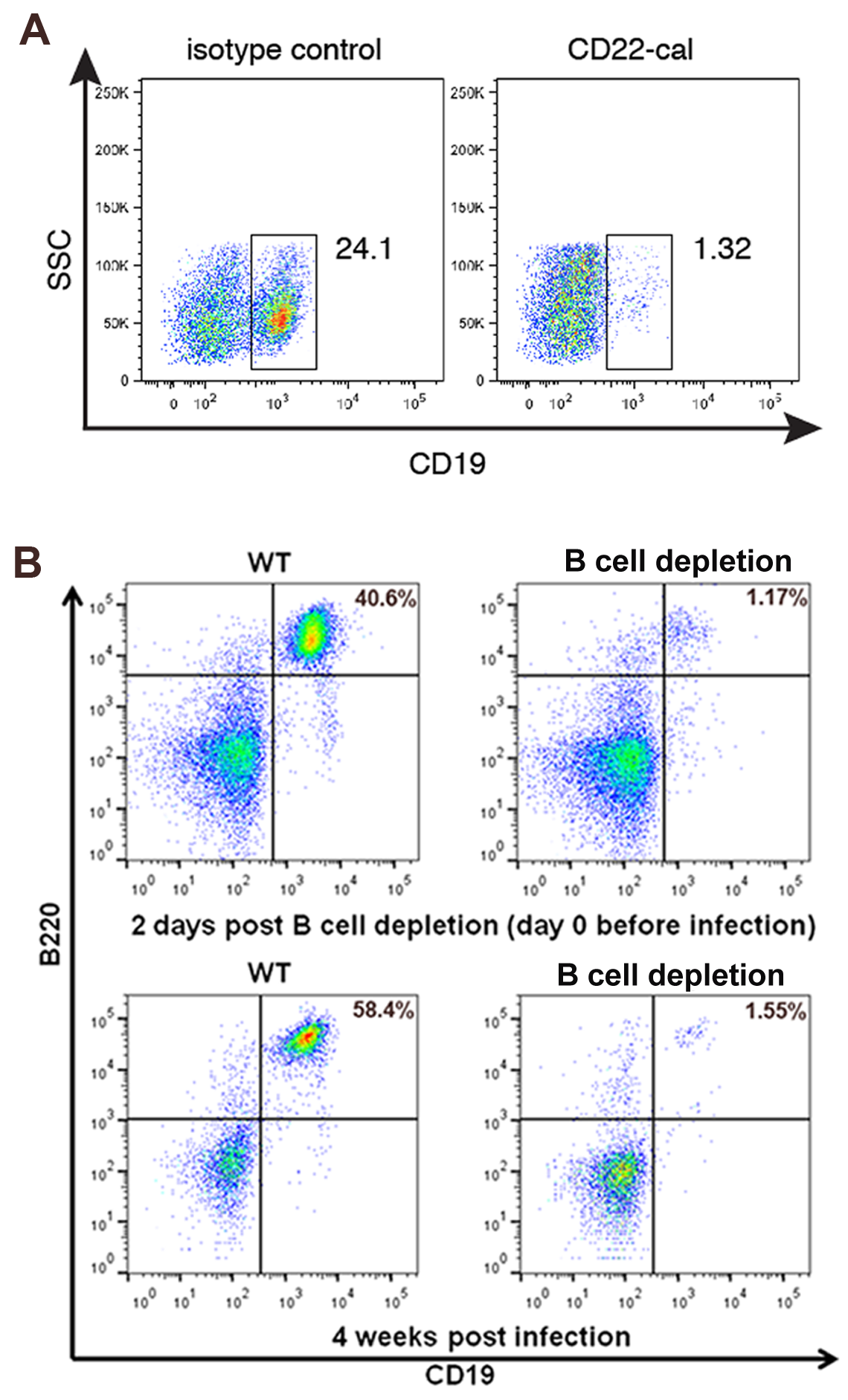

Supplement: Figure S1 — B cell depletion in C57BL/6 mice using CD22-cal and the 5D2 anti-CD20 Abs. A, Administration of CD22-cal according to the protocol described in Materials and Methods resulted in ∼95% depletion of CD19+ B cells in the lungs of M. tuberculosis-infected mice at day 21 p.i.. B, Administration of the 5D2 anti-CD20 mAb according to the protocol described in Materials and Methods led to effective B cell depletion (∼97% depletion) at the start of M. tuberculosis aerogenic infection of C57BL/6 mice that was maintained for the duration of the experiment (4 weeks p.i.). Results shown are representative of 2 independent experiments with 3 mice in each experimental group. WT: wild-type C57BL/6 mice treated with isotype control Abs. (TIF) [file ppat.1003472.s001.tif]
